# Lifestyle Modifications and Colorectal Cancer

**DOI:** 10.1007/s11888-013-0203-4

**Published:** 2014-01-12

**Authors:** Lukasz Durko, Ewa Malecka-Panas

**Affiliations:** Department of Digestive Tract Diseases, Medical University of Lodz, Kopcinskiego 22, 90-153 Lodz, Poland

**Keywords:** Colorectal cancer, Prevention, Lifestyle modification, Phytochemicals, Obesity, Physical activity

## Abstract

Many studies suggest that Western lifestyle and dietary factors may be responsible for the high incidence of colorectal cancer in industrialized countries. Consumption of high amounts of red and processed meat and low intake of fiber and multiple protective phytochemicals found in fruits, vegetables, and whole grains might be responsible for the high incidence of this neoplasm in the Western world. Additionally, obesity, lack of physical activity, tobacco and alcohol use, sleep deprivation, and other factors have been proven to further increase the risk of colorectal cancer. Identifying and understanding the mechanisms through which they impact colon carcinogenesis is needed for the introduction of protective lifestyle recommendations.

## Introduction

Colorectal cancer (CRC) is the second commonest neoplasm in women and the third commonest neoplasm in men. According to the World Health Organization GLOBOCAN database, in 2008 approximately 1.2 million new cases of CRC were diagnosed and 608,000 people died of CRC [1]. Despite screening programs designed for early detection of CRC having been introduced in many countries, the incidence of this neoplasm has not decreased, partly because of low compliance [2]. The occurrence of this disease varies approximately 25-fold in different world regions. The highest CRC incidence is noted in highly industrialized countries, whereas in developing countries CRC is not diagnosed so commonly [3]. Additionally, the number of new CRC cases increases in countries that undergo rapid economic transformations and adopt a Western lifestyle. This phenomenon occurred in eastern European countries, Asia, and some South American countries in the last three decades [4]. This observation strongly suggests that one of the key mechanisms of carcinogenesis of this neoplasm is associated with environmental factors [5, 6]. Therefore, identifying those mechanisms seems to be the most important goal in avoiding risk factors and developing prophylactic strategies leading to decreased incidence of CRC.

Many environmental factors have been studied, with imbalanced diet, alcohol consumption, tobacco use, obesity, lack of physical activity, and sleep deprivation being identified as the main CRC etiological factors [7]. Alimentary factors seem to have a major role, and the highest CRC incidence is noted in countries where the diet consists mainly of red and processed meat with low amounts of fiber sources, i.e., fruits, vegetables, and grains. On the other hand, some dietary factors have been proven to have chemopreventive properties [7]. Many molecular mechanisms of environmental factors promoting carcinogenesis have been discovered recently, which gives hope that it will be possible to reduce the incidence of CRC.

## Dietary Factors

### Fiber-Rich Foods

One of the earliest hypotheses that indicated diet as a risk factor for CRC was based on the observation that in the areas of low incidence of this neoplasm (Africa, Asia) the consumption of fiber is greater than in the Western world [8]. A high-fiber diet consists mainly of vegetables, fruits, and grains. Their presence in meals contributes to decreased transit time through the gastrointestinal tract, dilution of colonic contents, and enhancement of bacterial fermentation, which leads to increased production of short-chain fatty acids (acetate, propionate, and butyrate) [9]. Moreover, these substances were found to induce apoptosis in CRC cells in rats [10]. Dietary fiber has also been proved to have an anti-inflammatory function, decreasing the production of IL-6 and TNFα and also cyclooxygenase 2 (COX-2) and inducible nitric oxide synthase (iNOS) gene expression [11, 12]. Additionally short-chain fatty acids interfere with numerous regulators of the cell cycle, proliferation, and apoptosis, such as β-catenin, p53, p21, Bax, and caspase 3 genes in an animal model of CRC [13]. A meta-analysis based on 13 retrospective cohort studies presented by Howe et al. [14] in 1992 indicated that the risk of CRC is reduced by approximately 50 % in individuals who consume a high-fiber diet in comparison with the general population. However, prospective cohort studies performed in the USA did not confirm that fruit, vegetable, and grain intake decreases the risk of CRC [15, 16].

Another theory for the protective role of high-fiber diet indicates that such meals contain anticancer phytochemicals such as polyphenols, carotenoids, terpenes, and thioethers that interfere with cancer intracellular signaling cascades [17]. The presence of these substances is mainly observed in cruciferous and green leafy vegetables, onion, garlic, and citrus fruits. The above-mentioned studies presenting no chemopreventive role of high-fiber diet in CRC were conducted in the USA, where the most consumed vegetables are potatoes, lettuce, and tomatoes, which do not consist of many protective phytochemicals [18, 19]. The European Prospective Investigation into Cancer and Nutrition (EPIC) analyzed fiber intake and incidence of CRC in more than 500,000 individuals, and showed that doubling total fiber consumption decreases the incidence of this neoplasm by 40 % [20]. Moreover, in the observed population the incidence of CRC in individuals consuming high amounts of fruits and vegetables was 25 % lower than that in the remaining group [21].

The data presented clearly indicate that the risk of CRC can be decreased by consumption of plant-derived meals rich not only in fiber but also in protective phytochemicals. Many authors suggest that consumption of fruits and legumes, especially cruciferous and green leafy vegetables, might impact the prevalence of this disease.

### B Vitamins

Foods rich in fiber, beside containing protective phytochemicals, are often rich in numerous vitamins. The B vitamins have received special attention mostly because of their role in DNA synthesis, repair, and methylation [22]. Recent molecular in vitro studies also showed that low folate levels enhance invasiveness of colon cancer cells mediated by activation of the Hedgehog Shh signaling pathway through promoter hypomethylation and stimulation of the nuclear factor κB (NF-κB) pathway [23].

Most studies have shown that high intake of folic acid (vitamin B_9_), which is found in dark-green vegetables, reduces the risk of CRC and adenomas. This association is, however, correct only for natural dietary folic acid intake and not for its pharmacological supplementation [24]. Cole et al. [25] in a randomized clinical trial found that folic acid supplementation at a dosage of 1 mg/day is harmful, causing a 2.3-fold increase in the total number of colonic adenomas and a 1.7-fold increased risk of advanced colonic adenomas. Cole et al. suggested that only physiological levels of folic acid play a protective role, whereas intense supplementation may lead to progression of small preexisting adenomas. Additionally, supplementation of folic acid in high dosages (1,000 μg/day) appears to increase the risk of neoplasms, especially in the elderly population [26].

The chemopreventive function of folic acid is partially explained by its abilities to suppress the loss of heterozygosity of the tumor suppressor gene *DCC* (deleted in CRC) and to attenuate the *EGFR* gene by the folic acid metabolite 5-methyltetrahydrofolate [27].

Vitamin B_6_, or pyridoxal phosphate, which is also found in numerous fruits, vegetables, and grains, is another important protective anticancer substance. Larson et al. [28] presented a meta-analysis of prospective studies, and showed that the risk of CRC decreases by 49 % for every 100 pmol/mL increase of pyridoxal phosphate concentration in the serum.

### Red Meat and Processed Meat Intake

The consumption of red meat (beef, pork, lamb, veal, and mutton) is high in developed countries. A recent study by Daniel et al. [29] showed that its average daily intake in the USA is 128 g. Most consumed meat undergoes thermal processing or earlier preservation [29]. A recent meta-analysis of 21 prospective large-cohort studies indicates that the risk of CRC increases approximately linearly with higher intake of red and processed meat: the risk increased by 14 % for every daily 100-g increase of red meat intake [30••].

There are a few possible mechanisms that might explain that fact. One of the important factor leading to carcinogenesis might be increased intake of heme, which is present in red meat in high concentrations. Poultry and fish, considered as white meat, have tenfold lower amounts of this porphyrin pigment. Dietary heme is degraded in the small intestine by the enzyme heme oxygenase 1, releasing free ferrous iron [31]. Iron promotes the production of reactive oxygen species (ROS), especially H_2_O_2_, which induces genetic mutations and expression of numerous cytokines (IL-6, IL-8, TNFα, NF-κB), leading to increased cytotoxicity and stimulation of an inflammatory response [32].

Another mechanism that may be responsible for CRC development is connected to malondialdehyde, which is a product of lipid peroxidation. Malondialdehyde is a carcinogen, the action of which leads to DNA degeneration, causing single-strand breaks and double-strand breaks [33]. Additionally, during digestion of red meats, *N*-nitroso compounds are created, resulting in the formation of DNA complexes by binding them through telomere stabilizing proteins (TRF2) [33]. Consumption of red meat leads also to excess production of oxysterols and aldehydes, which induces transforming growth factor β expression and promotes cell proliferation [34].

Processing of red meat, especially by frying or grilling at high temperatures, causes degradation of muscle creatinine and amino acids, resulting in the formation of numerous carcinogenic heterocyclic amines [35, 36].

The data presented indicate that consuming poultry, fish, or legumes instead of red meat as the main source of proteins would be beneficial in decreasing the risk of CRC. Additionally, avoiding meat processed at high temperatures might also have a protective effect.

### Grape Seed Extract

Grape seed extract (GSE) is a mixture of polyphenols, mainly proanthocyanidins. Molecular in vivo studies have shown that it inhibits aberrant β-catenin, cyclin D1, and c-myc expression, preventing cycle cell disruption. Additionally, GSE reduces expression of iNOS and COX-2, decreasing oxidative cellular stress [37]. Derry et al. [38] performed in vitro studies on CRC cell lines, and showed that GSE induces their apoptosis, mainly due to activation of casapses 3, 8, and 9 and also generation of ROS. Moreover the proapoptotic function of GSE was limited only to cancer cells and there was no effect in normal colonocytes.

However, the available data are based only on animal models and in vitro studies on human CRC cell lines, but GSE supplementation seems to hold promise for chemoprevention.

### Silibinin

Silibinin is a falvonolignan extracted from milk thistle (*Sylibum marianum*). It is the main component of silymarin—a supportive medication used in the treatment of many liver diseases. Several in vitro and in vivo studies have shown its chemopreventive role in skin cancer, lung cancer, prostate cancer, bladder cancer, and CRC by targeting DNA mutation mechanisms, proliferation, metastatic signaling, and inflammation processes [39]. Silibinin was found to inhibit the Wnt/β-catenin pathway, causing further decrease of cyclin D1 and c-myc expression [39, 40]. Moreover, silibinin deactivates antiapoptotic proteins such as BCL-2, MCL-1, X-linked inhibitor of apoptosis protein, and survivin [37]. On the other hand, it upregulates transcription of the death receptors DR4 and DR5, promoting apoptosis [41]. A recent in vitro study on human CRC cell lines showed that silibinin inhibits TNFα activation of NF-κB. Furthermore, it decreases expression of COX-2 and iNOS. Additionally, silibinin causes endoplasmic reticulum stress and inhibits mitochondrial glucose uptake in CRC cells [42]. Importantly, silibinin is not toxic to normal colonic epithelium. Hoh et al. [43] conducted a study evaluating silibinin pharmacodynamics. Its levels were monitored in plasma, liver, and colonic tissue, and high concentrations of this substance were found in colonocytes in patients receiving this herbal supplement orally. This promising finding supports the need for further investigations on the chemopreventive properties of silibinin.

### Curcumin

Curcumin is a phenolic extract of the spice turmeric (*Curcuma longa*) with antioxidative potential used in ancient medicine. Numerous early-phase clinical trials have proved the safety and low systemic concentration of curcumin after oral admission in CRC [44, 45•]. Curcumin is effective in promoting apoptosis and inhibiting DNA mutations, cancer cell proliferation, metastasis, and inflammation. Curcumin upregulates glutathione *S*-transferases and induces an ROS concentration that leads to p21 protein upregulation, inhibiting cancer cell growth [46, 47]. Curcumin interferes with the mitogen-activated protein kinase (MAPK) pathway, leading to decreased production of TNFα and COX-2 and downregulation of NF-κB and IL-6 expression, preventing development of inflammation [48, 49]. Lower NF-κB levels also result in downregulation of c-myc, cyclin D1 and BCL-2 genes, modulating the cell cycle [50].

Interesting results were obtained in in vitro studies on the colon cancer cell line HT-29 incubated with the combination of curcumin and the COX-2 inhibitor sulindac sulfone. The effects of such treatment resulted in a synergistic inhibitory effect on cell growth, cell cycle arrest, and induction of apoptosis of cancer cells [51]. Similar results were found in rats treated with 1,2-dimethylhydrazine. The curcumin and sulindac sulfone treatment reduced the number of aberrant crypt foci by 75 % comparing with the control group [51]. This finding may be clinically important since addition of curcumin allows lower and less toxic sulindac sulfone doses to be used, resulting in safer treatment of CRC.

Another study evaluated the effect of Coltect (a dietary supplement containing curcumin) used together with 5-aminosalicylic acid in vivo on rats treated with 1,2-dimethylhydrazine. Coltect reduced the number of aberrant crypt foci similarly as 5-aminosalicylic acid by 40 %. The use of both substances resulted in a synergistic effect, reducing the number of aberrant crypt foci by 70 % [52].

Furthermore, curcumin promotes cancer cell apoptosis by inducing expression of proapoptotic proteins (Bax, Bim, Bak, Noxa) and inhibiting expression of antiapoptotic proteins (BCL-2, BCL-xl) [53]. Curcumin also plays a role in decreasing vascular endothelial growth factor (VEGF) and matrix metalloproteinase 9 expression, which prevents the formation of metastases [50]. Other recently presented data show that curcumin inhibits CRC growth in a murine model and in vitro by downregulating microRNA-21, which restores the function of the phosphatase and tensin homolog tumor suppressor gene [54]. Additionally, Patel et al. [55] have shown in in vitro studies that curcumin inhibits HER2 and insulin-like growth factor 1 (IGF-1) receptor expression in CRC cells that are resistant to 5-fluorouracil and oxaliplatin used as chemotherapy for this neoplasm.

Curcumin seems to be one of the most promising widely available chemopreventive agents which reduces CRC risk. However, studies focusing on the influence of dietary intake of meals rich in curcumin on the incidence of this CRC are required.

### Epigallocatechin 3-Gallate

Epigallocatechin 3-gallate (EGCG) is a polyphenol found in tea leaves of *Camellia sinensis*. Animal model studies suggest its chemopreventive function on various levels [53]. EGCG is a strong antioxidant, preventing ROS formation. Additionally, it decreases the expression of growth factors (epidermal growth factor, IGF-1, VEGF), blocking cancer cell proliferation and metastasis formation [56]. EGCG also blocks the cell cycle through downregulation of the MAPK/extracellular-signal-regulated kinase 1/2 and p21 signaling pathways [57]. Furthermore, it induces apoptotic abilities by upregulation of p53 and p21 [58]. However, a clinical phase II study examining the effectiveness of EGCG in the treatment of advanced ovarian cancer did not show any benefits of this substance, and further investigations in CRC and other tumors are needed [59].

## Probiotics

Intestinal microbial flora regulate multiple physiological functions related to cell proliferation, differentiation, and stimulation of immunity. The DNA mutations in CRC reduce the integrity of colonic epithelium, allowing bacterial translocation and further activation of the cytokines IL-1, IL-2, and IL-23 and stimulation of T_h_17 lymphocytes, which further promotes cell proliferation [60•]. Interestingly, extensive colonization with pathogenic *Fusobacterium* species, which was earlier described as a possible etiological factor for inflammatory bowel diseases, was discovered in CRC tumors [61]. Additionally, genotoxic strains of *Escherichia coli* leading to numerous DNA mutations are often cultured from CRC tissue [62].

Probiotics similarly impact the molecular mechanisms of cell growth and differentiation. Many in vitro and animal model studies have shown multiple possible pathways through which probiotics might prevent CRC. Their presence might competitively reduce the number of pathological colonic flora, reduce carcinogenic secondary bile acid production, and increase production of short-chain fatty acids, modulating the inflammatory response [63]. The type of mechanism depends on the microbial species. *Saccharomyces boulardi*, a yeast species, has antioxidant and anti-inflammatory effects. Polyamines produced by this species reduce the oxidative stress. Additionally, in vitro and animal model studies have shown that *S. boulardi* downregulates the MAPK/extracellular-signal-regulated kinase 1/2 signaling pathway, which might explain its potential antiproliferative effect [64]. Other probiotics have different potential anticancer mechanisms: *Bifidobacterium animalis* modulates IGF-1 expression, and *Bacillus polyfermenticus* suppresses epidermal growth factor receptor pathway signaling [65, 66]. However, the lack of convincing data from clinical studies does not allow probiotics to be recommended as chemopreventive agents in CRC.

## Alcohol

The risk of CRC and colonic adenomas is associated with alcohol consumption. Several prospective cohort studies have shown that consumption of more than 30 g of ethanol per day results in a multivariative risk of CRC of 1.16, whereas consumption of more than 45 g of ethanol per day increases that risk to 1.41 [67]. Metabolism of alcohol is based mainly on catalysis and oxidation by alcohol dehydrogenase, catalase, and cytochrome P450, resulting in the formation of acetaldehyde, which is a class 1 carcinogen and is responsible for chromosome damage [68•]. An in vitro study on ethanol and acetaldehyde genotoxicity showed a dose-dependent increase in the number of DNA strand breaks particularly in colonic mucosa cells and lymphocytes [69].

In addition to promoting carcinogen activity, long-term alcohol consumption decreases the absorption of group B vitamins (B_1_, B_2_, B_12_, folic acid), which causes increased cell vulnerability to oxidative stress [68•]. Increased ROS levels induce the NADPH oxidase cascade, leading to activation of the phosphoinositide 3-kinase (PI3K)/AKT and VEGF pathway, which promotes proliferation and metastasis [70].

Additionally, alcohol blocks expression of the enzyme cytochrome P450 2E1, which is involved in vitamin A synthesis. The low vitamin A levels decrease the expression of activator protein 1—a transcription factor which controls cell differentiation and proliferation [68•].

Therefore, it is reasonable to recommend the reduction of alcohol consumption, which reduces the risk of CRC development.

## Tobacco

Many studies have shown that tobacco use increases the risk of CRC and colonic adenomas [70]. The multicenter, prospective study EPIC, conducted on 465,879 individuals with a mean follow-up of 8.7 years, shows that such risk is observed in both past and present smokers (relative risk 1.21) and most CRC tumors in smokers are located on the right side of the colon [71]. The main carcinogens found in tobacco smoke are aromatic amines, nitrosamines, heterocyclic amines, and polycyclic aromatic hydrocarbons. These substances undergo metabolism through cytochromes P450, leading to the formation of aberrant DNA and further gene mutation (*KRAS*, *BRAF*, *MYC*) [71]. Nitrosamines have the ability to activate and bind to nicotinic acetylcholine receptors, which results in an increase of intracellular ROS concentrations. The oxidative stress leads to activation of the NF-κB and COX-2 inflammatory pathways and also promotes the MAPK proliferative signaling cascade [72]. Nicotine activates β-adrenoreceptors, which triggers inflammatory and metastatic signaling through the COX-2, metalloproteinase 2, and VEGF pathways [73].

There is no doubt that avoiding tobacco use decreases the risk of CRC and other neoplasms and also prevents the development of cardiovascular disease, pulmonary disease, and other diseases.

## Obesity

Many studies have indicated that obesity, defined as body mass index (BMI) exceeding 25 kg/m^2^, is a risk factor for multiple neoplasms. This has been confirmed by a recent meta-analysis by Renehan et al. [74]. Another systematic review concentrating only on CRC and adenoma incidence indicated their higher prevalence in obese individuals, particularly in men. Compared with BMI below 23.0 kg/m^2^, for BMI of 23.0–24.9, 25.0–27.4, and 27.5–29.9 kg/m^2^ and BMI greater than 30 kg/m^2^, the risk of CRC was 14, 19, 24, and 41 %, respectively [75••, 76]. Moreover, abdominal visceral adipose tissue volume was proposed to be a more adequate risk factor for CRC than BMI or waist circumference, explaining the higher prevalence of this neoplasm in obese men than in obese women [77••].

The mechanisms for increased cancer incidence in obese individuals have not been fully elucidated. Adipose tissue is considered as a metabolically active organ releasing numerous hormones and cytokines and stimulating T cells that promote low-intensity chronic inflammation and insulin resistance [78]. Additionally, increased serum triglyceride concentration in obese individuals further enhances this process [79]. Hyperinsulinemia, which is the result of insulin resistance, contributes, together with IGF-1, to increased cell proliferation and potential carcinogenesis [80]. Moreover, adipose tissue secretes adipohormones such as leptin, adiponectin, and resistin which contribute to promotion of inflammation and carcinogenesis [81]. Booth et al. [82] have shown that serum leptin and adiponectin levels were elevated in CRC patients and patients with colonic adenomas compared with healthy individuals.

The data presented suggest that body mass reduction, which decreases chronic inflammation, glucose intolerance, and dyslipidemia, might impact CRC incidence and prevent the development of multiple obesity-related diseases.

## Physical Activity

Many chronic diseases, including cardiovascular, pulmonary, and musculoskeletal diseases, type 2 diabetes, and many types of cancer, are associated with insufficient physical activity [81]. A meta-analysis by Wolin et al. [83] indicates that most physically active individuals have 24 % lower risk of CRC development than those who have a sedentary lifestyle. The same authors conducted another systematic review, showing 16 % reduction in the incidence of colonic adenomas and 35 % reduction in the incidence of large colonic polyps in the physically active group [84••].

The key mechanisms that explain the protective role of physical activity focus on increased insulin sensitivity, lower insulin levels, decreased body mass, and decreased adipose tissue volume, leading to reduction of chronic inflammation [85].

Current guidelines of the American Institute of Cancer Research recommend a minimum of 60 min of moderate or 30 min of intense physical activity daily to promote health [86]. However, excessive exercise might be harmful, leading to increased oxidative stress and consequent DNA damage. Such observations were not found during moderate exercise [86].

Ashgar et al. [87] found in rat models that physical activity induces superoxide dismutase activity, which through the transcription factor Nrf2 initiates the expression of detoxifying enzymes such as glutathione *S*-transferases.

Another protective mechanism induced by physical activity focuses on the role of the adipohormone leptin, which induces proliferative signaling pathways through activation of the MAPK and PI3K/AKT cascades. Moderate exercise was found to decrease serum leptin levels [88]. Additionally, high adipose tissue volume induces low-grade inflammation, mainly through TNFα overexpression. Such chronic inflammation is believed to promote carcinogenesis [89]. Physical activity promotes the production of IL-6 and decreases the expression of iNOS and TNFα both in plasma and in colonic mucosa, leading to enhanced immunity and also increased lipolysis in adipose tissue [90, 91]. Moreover, Ju at el. [92] showed in an animal model that physical activity inhibits the IGF-1/insulin-like growth factor binding protein 3 expression and abnormal β-catenin signaling pathway. The aberrant β-catenin pathway induces further modulation of oncogenic genes such as c-myc and VEGF expression; therefore, maintaining the equilibrium of this cascade might be an important anticancer mechanism.

The clinical and molecular studies presented clearly indicate that physical activity leads to metabolic transformations resulting in a decrease of CRC risk. Additionally, weight reduction induced by physical exercise in overweight and obese individuals might also be protective.

## Circadian Rhythm

Some authors have investigated carcinogenesis related to chronic sleep deprivation or disruption of circadian rhythm. Hrushesky et al. [93] have shown that nightshift workers, both men and women, have 50 % risk of developing CRC.

It has been proven that cell proliferation, differentiation, apoptosis, and DNA repair mechanisms have different day and night activities [93]. Some of the enzymes expressed in rhythmic manner are cytochromes P450 involved in detoxication mechanisms. Another such gene is the p21/Weel gene, which regulates the cell cycle, DNA replication, and mitosis [94]. Additionally, so-called circadian clock genes have been described and studied recently: *PER1*, *PER2*, *PER3*, and *CLOCK* [95]. Mice with a silenced *Per2* gene showed no p53 protein activity, which resulted in dysfunction of the DNA repair and apoptosis pathways [95]. Moreover *PER2* expression was shown to be high in cancer cells and to promote the c-myc, cyclin D, β-catenin, and VEGF signaling pathways [96].

The data presented suggest that maintaining a regular and adequate daily amount of sleep contributes to CRC prevention.

## Other Factors

Numerous studies have indicated that oxidative stress is one of the key promoters of carcinogenesis, as presented earlier. Various protective substances have been examined. Vitamin E (α-tocopherol) has been shown to decrease colonic epithelial DNA damage evoked by bleomycin in in vitro studies [97]. Another study indicated that gene mutations in colonic mucosa induced by nickel could be attenuated by quercetin, a flavonoid obtained from many fruits and vegetables [98].

There are a variety of medications with a chemopreventive role in CRC development, but describing their function is beyond the scope of this article. However, recent data on the effects of aspirin intake in prevention of CRC are noteworthy. Aspirin inhibits COX-2, reducing the oxidative stress and limiting gene mutations. Previous studies have shown that aspirin at dosages of 300 mg or more per day reduces the risk of CRC. However, the numerous side effects of this drug excluded it from chemopreventive recommendations [98]. Interestingly, recent studies have shown that aspirin taken after the diagnosis of CRC in patients with *PI3K* mutation decreases tumor growth and improves survival [99, 100].

## Conclusion

The studies presented indicate that diet and lifestyle habits could impact the incidence of CRC. Therefore, it is reasonable to suggest general recommendations for lifestyle modifications that would decrease the risk of CRC. Replacing red and processed meat, highly saturated fats, refined starches, and sugars with fish, poultry, vegetables, fruits, and grains as sources of polyunsaturated fats, carbohydrates, and proteins might lower the risk of this neoplasm. Additionally avoiding smoking and alcohol consumption, reducing obesity, especially by physical activity, and maintaining adequate sleep patterns also appear to be very beneficial. Moreover, introducing a chemopreventive agent such as curcumin, GSE, EGCG, or silibinin might further lower the incidence of CRC.

Following those regulations would not only decrease the risk of CRC but would also improve overall health status and decrease the incidence of other neoplasms and cardiovascular disease, pulmonary disease, musculoskeletal disease, and multiple other diseases.
